# Retinal Degeneration and Regeneration—Lessons From Fishes and Amphibians

**DOI:** 10.1007/s40139-017-0127-9

**Published:** 2017-01-25

**Authors:** Divya Ail, Muriel Perron

**Affiliations:** 1 Paris-Saclay Institute of Neuroscience, CNRS, Univ. Paris-Sud, Université Paris-Saclay, Orsay, France; 2 Centre d’Etude et de Recherche Thérapeutique en Ophtalmologie, Retina France, Orsay, France

**Keywords:** Retinal degeneration, Retinal regeneration, Retinal stem cells, Müller glial cells, Ciliary marginal zone, Retinal pigment epithelium

## Abstract

**Purpose of Review:**

Retinal degenerative diseases have immense socio-economic impact. Studying animal models that recapitulate human eye pathologies aids in understanding the pathogenesis of diseases and allows for the discovery of novel therapeutic strategies. Some non-mammalian species are known to have remarkable regenerative abilities and may provide the basis to develop strategies to stimulate self-repair in patients suffering from these retinal diseases.

**Recent Findings:**

Non-mammalian organisms, such as zebrafish and *Xenopus*, have become attractive model systems to study retinal diseases. Additionally, many fish and amphibian models of retinal cell ablation and cell lineage analysis have been developed to study regeneration. These investigations highlighted several cellular sources for retinal repair in different fish and amphibian species. Moreover, major differences in repair mechanisms have been reported in these animal models.

**Summary:**

This review aims to emphasize first on the importance of zebrafish and *Xenopus* models in studying the pathogenesis of retinal diseases and, second, on the different modes of regeneration processes in these model organisms.

## Introduction

Retinal diseases resulting in impaired vision and blindness both severely compromise the quality of life of the individual and have proven to be socio-economic burdens. Hence, a substantial amount of research efforts in the field are directed towards understanding the disease mechanisms and developing therapies to combat these disorders.

The clinical trial success of an AAV-mediated gene therapy to correct the RPE65 mutation causing Leber congenital amarousis (LCA) [1, 2] is encouraging the use of this strategy for other diseases with distinct genetic causes. However, such therapies do not hold much potential in the case of Retinitis pigmentosa (RP), wherein more than 200 different mutations affecting 50 different genes have been linked to the disease [3]. Another complex, multi-factorial disease is age-related macular degeneration (AMD), which has factors such as age and lifestyle [4, 5] in addition to genes [6] that contribute to the onset of symptoms, progression of the disease, and response to the limited therapies. AMD is classified into wet or dry depending on the occurrence or lack of neovascularization, respectively. The randomly formed blood vessels leak blood and fluids into the retina resulting in rapid and severe retinal damage. The most widely accepted anti-vascular endothelial growth factor (VEGF) therapy is provided only for wet AMD patients who account for 15% of the total AMD cases [7, 8]. Several therapies are provided to delay the onset or slow down the progression of the disease, with varying degrees of success [9]. However, a therapy for those suffering from dry AMD still remains an unmet medical need. Cell replacement therapies attempt to replace lost neuronal cells of the retina with retinal progenitors or pre-differentiated cells [10, 11]. Attempts to restore rod function and vision in murine models of retinal degeneration proved successful [12]. This further encouraged cell replacement studies, using cells derived from 3D cultures of embryonic cells [13] as well as the more recent transplantation of human embryonic stem cell (hESC)-derived retinal tissue into primate models of retinal degeneration [14•]. Retinal pigment epithelium (RPE) cells have been successfully replaced by cells originating from ESCs [15], bone marrow-derived hematopoietic stem cells (BMHSCs) [16], or induced pluripotent stem cells (iPSCs) [17, 18]. Although some of these therapies have slowly made the transition from the research laboratories to clinical trials [19–20, 21•], the ability of the transplanted cells to integrate in the local environment, make the right connections, and restore vision will eventually determine their efficacy as actual treatments. Unexpectedly, two recent studies showed that stem cell-derived photoreceptor progenitors transplanted into the murine retina do not actually integrate within the retina as previously suggested. These transplanted cells stay in the subretinal space at the site of injection and are involved in an exchange of cytoplasmic material with the host photoreceptors [22•, 23•]. On the other end of the spectrum, technological advances in the field of retinal prosthesis have led to the development of retinal implants that allow partial restoration of low-resolution vision in some patients, which enables them to perceive light and discern large objects. These implants work by electrically stimulating the surviving neurons in the diseased retina [24].

All these multi-faceted attempts to fight visual impairment and loss are owing to the fact that the mammalian retina is incapable of repairing or regenerating itself. Once the mammalian retinal neurons are lost due to injury or disease, they cannot be restored by endogenous mechanisms. However, there are many species including zebrafish and *Xenopus* that are known to have remarkable regenerative abilities. Understanding these mechanisms and manipulating them could allow for the development of an alternative therapeutic strategy aimed at triggering self-repair in the mammalian retina.

**Fig. 1:**
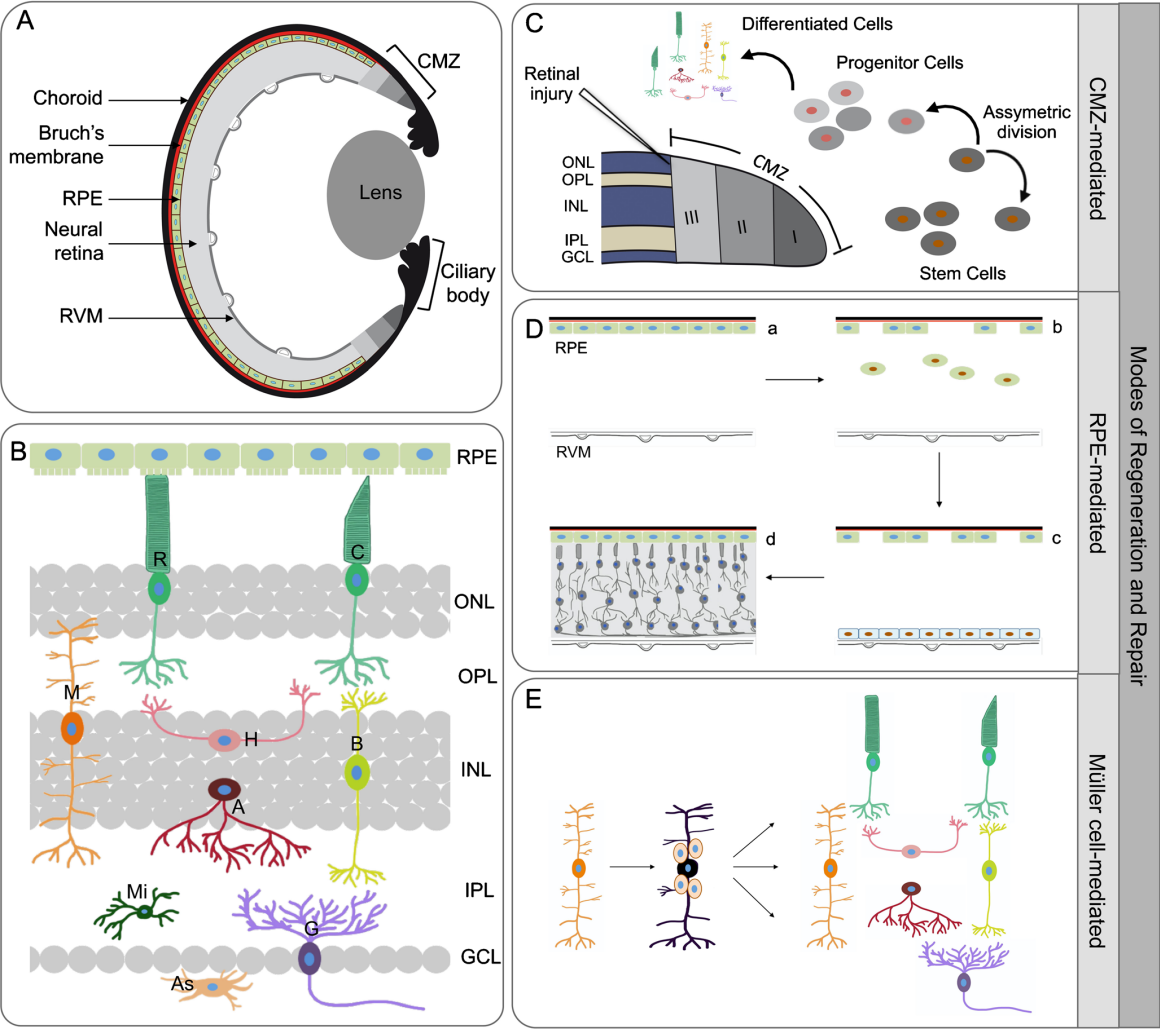
**a** Structure of the retina. Schematic representation of a cross section of the zebrafish or *Xenopus* eye showing the ciliary marginal zone (CMZ), retinal pigment epithelium (RPE), neural retina, choroid, Bruch’s membrane, and the retinal vascular membrane (RVM). **b** Cell types of the retina. The retina is composed of different cell types: the nuclei of the two types of photoreceptors, rods (R) and cones (C), form the outer nuclear layer (ONL), whereas the Müller cells (M), horizontal cells (H), bipolar cells (B), and amacrine cells (A) are present in the inner nuclear layer (INL), and the ganglion cells (G) in the ganglion cell layer (GCL). The axons of these neurons and glial cells form synaptic connections in the outer and inner plexiform layers (OPL and IPL). The astrocytes (As) are located near the blood vessels whereas the microglia (Mi) are mostly located in the plexiform layers but can be distributed through the different layers. **c–e** Modes of regeneration and repair. CMZ-mediated (**c**): In the constantly growing retinas of zebrafish and *Xenopus*, the spatial cellular gradient in the CMZ recapitulates embryonic retinogenesis with zone I, the most peripheral part of the CMZ, where stem cells reside, zone II encompassing retinal progenitor cells, and zone III consisting of late retinal progenitors including post-mitotic retinoblasts. The stem cells divide asymmetrically to self-renew and generate one progenitor cell, and this mode of asymmetric division is retained even in the case of retinal injury. RPE-mediated (**d**): In *Xenopus* following partial retinectomy, wherein the RPE and RVM are left intact (*a*), a subset of RPE cells (*green oval cells*) detach from the Bruch’s membrane (in *red*) and migrate to the RVM (*b*). When they adhere to the RVM and form a distinct layer of cells (*blue cells*), they start proliferating (*c*) and regenerate the whole neural retina (*d*), while RPE cells that remained attached to the Bruch’s membrane renew the RPE layer (*d*). Müller-cell mediated (**e**): In zebrafish upon retinal injury, a subset of Müller cells (*orange*) undergo asymmetric division (*black*) to renew themselves and generate multipotent progenitor cells that can actively divide and regenerate all major retinal cell types (color figure online)

The mammalian retina is composed of stratified layers of five different types of neurons. Photoreceptors (rods and cones) are present in the outer nuclear layer (ONL) and form connections with horizontal cells, bipolar cells, and amacrine cells present in the inner nuclear layer (INL), which further connect to the ganglion cells in the ganglion cell layer (GCL). There are three types of glial cells in the retina, Müller cells, astrocytes, and microglia. The Müller cells originate from the same retinal progenitor as all retinal neurons [25], and they span all the layers of the retina making connections with and providing support to all the cell types. A single-cell layer of epithelial cells called RPE is present between the choroid and the outer segments of the photoreceptors (Fig. 1a, b). Major blinding diseases of the retina, such as AMD or RP, result from the direct loss of photoreceptors or follow the loss of RPE cells.

In addition to these cell types, the retinas of zebrafish and *Xenopus* have a region at the peripheral part of the retina composed of stem cells and progenitor cells that generate retinal cells in the larvae and adult eye. This region is called the ciliary margin zone (CMZ) and is composed of cells that are present in a spatial gradient: the extreme edge of the CMZ is occupied by stem cells, followed by committed proliferating retinal progenitors and finally post-mitotic progenitors [26–28] (Fig. 1a, c).

## Zebrafish and *Xenopus* Models for Degeneration and Regeneration Studies

### Models to Study Retinal Degenerative Diseases

Often retinal dystrophies have a genetic cause, and the onset and progression of the disease is gradual. Hence, attempts are being made to reproduce disease mutations in animal models to understand the pathogenic mechanisms of degeneration. Comprehensive reviews on the use of zebrafish models to study a broad range of retinal diseases have recently been published [29•, 30–31]. Here, we provide a few recent examples, highlighting the relevance of these models to study human retinal dystrophies. There are several models for retinal diseases affecting photoreceptor survival in zebrafish, wherein genes crucial for phototransduction have been mutated. For instance, mutation in the cone phosphodiesterase α-subunit gene (*pde6c*) causes cone degeneration followed by rod cell death, as observed in humans [32]. This zebrafish model allowed to better understand the underlying mechanism of cone and rod cell death and thus to propose that a combination therapy intervention in patients, directed at the different cell death mechanisms, could provide effective treatment strategies [33]. Many zebrafish mutants with photoreceptor degeneration carry mutations in cilia-related genes, which are important for proper intracellular trafficking. These models recapitulate various retinal ciliopathies in humans, such as the Bardet–Biedl syndrome (BBS) or the Joubert syndrome. Three recent reports exemplify the usefulness of zebrafish models to provide phenotype–genotype correlations or to discover novel genetic interactions related to retinal ciliopathies: a knockdown approach in zebrafish revealed a ciliary function for *vps15*, a gene in which mutations are found in patients with a ciliopathy [34•]; by overexpressing in zebrafish mutated forms of the *C8ORF37* gene, encoding a cilia protein, it was shown that *C8ORF37* variants cause BBS [35•]; the zebrafish mutant for *arl13b*, a gene that causes the classical form of Joubert syndrome, revealed that Arl13b genetically and physically interacts with the planar cell polarity protein (PCP) component Vangl2 and that this association is important for normal photoreceptor structure [36]. The feasibility of large-scale screens is clearly an advantage of using zebrafish as an animal model for human diseases. As an example, a whole-genome siRNA-based functional genomics screen was used to identify genes whose activity is required for ciliogenesis and/or cilia maintenance. This approach allowed the identification of a collection of ciliopathy genes and brought insights into pathogenic mechanisms of ciliopathies [37•]

Angiogenesis significantly contributes to the progression of retinal diseases such as AMD and diabetic retinopathy. Some zebrafish models have been generated to study retinal pathological angiogenesis. For instance, neovascularization is observed in zebrafish subjected to hypoxia or in von Hippel–Lindau (VHL) tumor suppressor mutants [38–40]. Immersing zebrafish in glucose solution induces hyperglycemia and serves as a model for diabetic retinopathy [41, 42]. Interestingly, it was shown that vascular changes are not sufficient to induce cone photoreceptor dysfunction, suggesting that the vascular and neuronal complications in diabetic retinopathy can arise independently. All these models for pathological angiogenesis and vascular retinopathies offer cost-effective ways to screen in vivo for efficient novel anti-angiogenic drugs [43].

Although *Xenopus* is not as genetically amenable as zebrafish, it has nonetheless emerged as a relevant model to study retinal diseases [44, 45]. Fourier domain optical coherence tomography (FD-OCT), a promising non-invasive imaging technique, proved to be efficient for monitoring in vivo the time course of retinal degeneration in *Xenopus* larvae [46]. Tam et al. generated a model for RP by causing a mutation in the *rhodopsin* gene (P23H), which is known to cause RP in humans. It was suggested that the P23H mutant protein is misfolded and retained in the endoplasmic reticulum (ER), leading to ER stress and ultimately causing rod death [47]. Interestingly, when these tadpoles were reared in the dark, the degeneration was rescued [48]. The authors thus suggest that protecting RP patients (with mutations in the N terminus of rhodopsin) from light could be of therapeutic value. Recently, it was reported that light-induced retinal degeneration caused by P23H rhodopsin occurs via cell death by autophagy [49•], supporting the hypothesis that multiple cell death mechanisms cause retinal degeneration [50]. Transgenic *Xenopus* expressing rhodopsin glycosylation mutants (T4K and T17M) exhibit light-exacerbated retinal degeneration, as in humans [51]. These *Xenopus* models helped demonstrate a novel pathogenic mechanism in which glycosylation-deficient rhodopsins become destabilized by light activation, leading to photoreceptor degeneration. Transgenic *Xenopus* models expressing mutated rhodopsin that do not lead to degeneration, but are known to cause autosomal dominant congenital night blindness (CNB) in humans, have also contributed to bring insights into the molecular mechanisms of the disease. The authors propose that rhodopsin mutations cause CNB as a result of persistent signaling by the constitutively active opsin [52]. Stargardt-like macular dystrophy is a juvenile macular degeneration caused by mutations in the *elongation of very long-chain fatty acids 4* (*ELOVL4*) gene [53]. A *Xenopus* transgenic model of this disease, overexpressing dominant negative ELOVL4 variants, has been generated [54]. Although normally present in the inner segments, the truncated protein was shown to be mislocalized to the Golgi compartments or the inner segments. This mislocalization was hypothesized to be responsible for the alteration in photoreceptor outer segment structure and function, resulting in degeneration [54].

### Models to Study Retinal Regeneration

Retinal regenerative studies in zebrafish benefit from a variety of injury paradigms: chemical lesions, physical or genetic methods of ablation, and light or thermal injury. Physical methods, such as surgical removal or needle incision, damage neurons in all retinal layers [55, 56]. Intravitreal injection of ouabain, a plant-derived Na/K-ATPase inhibitor, causes retinal degeneration in a dose-dependent manner in zebrafish. It affects either all layers or more selectively the GCL and INL without damaging photoreceptors [57]. To selectively damage neurons in the INL and GCL, NMDA-mediated neurotoxicity has also been used [58•].

To specifically cause photoreceptor loss, there are two widely used light damage paradigms [59–64]. One uses constant bright light, primarily damaging rod photoreceptors, whereas the other uses an exposure to extremely intense ultraviolet (UV) light for a short time period, targeting both rods and cones. More recently, a new light lesion paradigm was designed where light is focused through a microscope onto the retina of an immobilized fish. Such focused light lesion has the advantage of creating a locally restricted area of damage [65]. Local thermal lesions using heated copper wire also allows to locally limit retinal damage, affecting solely the RPE and underlying photoreceptors in the adult zebrafish retina [66]. Another novel paradigm to study photoreceptor regeneration was established based on optical coherence tomography-guided laser photocoagulation, which also induces localized lesions in the outer retina [67]. *N*-Methyl-*N*-nitrosourea (MNU) is an alkylating agent well known for inducing photoreceptor damage in rodents [68]. A similar MNU-induced model has recently been developed in zebrafish, leading to specific rod photoreceptor degeneration, without any significant cone loss [69, 70]. This MNU-induced damage is less invasive than other chemical methods as it does not require intravitreal injections, but can be dissolved in the tank water of the zebrafish. Finally, transgenic zebrafish have been engineered to allow conditional ablation of rods [71] or of single cone subtypes [72], using the nitroreductase–metronidazole (NTR–MTZ) system [73]. These transgenic zebrafish express the *ntr* gene under the control of a specific promoter. When the fishes are treated with MTZ, the NTR converts it into a cytotoxic compound that does not diffuse to neighboring cells, thereby resulting in targeted ablation of the NTR-expressing cells. This process is reversible, so removing the tadpoles from the MTZ solution allows examination of the regeneration process.

Regeneration studies in *Xenopus* have extensively relied on mechanical approaches affecting all retinal layers, such as complete retinectomy [74–77, 78•, 79], partial retinal excision [80, 81], or simple needle incision (our unpublished data). Several conditional cell ablation transgenic *Xenopus* models have also been engineered. For instance, a modified inducible procaspase 9 (iCasp9) containing binding domains for the pharmacological compound AP20187 was specifically expressed in the rod photoreceptors of *Xenopus laevis*. Upon addition of AP20187, dimerization and autoactivation of iCasp9 result in rod cell death [82]. Another inducible model, using the NTR–MTZ system described above, has also been used to conditionally and reversibly ablate rod photoreceptors ( [81] and our unpublished data).

## Modes of Regeneration and Repair

The structure, the cell types, and the function of the retina are largely conserved among vertebrates, yet the regenerative ability varies tremendously among groups and has been lost through evolution. Furthermore, even species that regenerate displaying major differences in the cellular and molecular mechanisms used to do so. In the various lesion paradigms described above, the damaged retina regenerates within several days to weeks in zebrafish and *Xenopus*. Described below are the different modes of retinal regeneration employed by these species.

### From the CMZ

Retinas of teleost fish and amphibians are continuously growing throughout their lifetime owing to retinal stem and progenitor cells located in the CMZ at the rim of the retina [83–87]. Recent studies showed the presence of a CMZ-like region in cartilaginous fishes [88, 89], as well as in a reptilian, the painted turtle [90]. However, no CMZ was observed in few specimens of adult lizards and snakes [90]. The CMZ seems to have gradually diminished during vertebrate evolution, being present in the post-hatched bird but not in the adult and absent in mammals [91, 92]. Interestingly, however, a CMZ-like zone was observed in self-organizing human optic cups derived from hESCs, at the junction of the RPE and the neural retina [93•].

The presence of true self-renewing and multipotent neural stem cells in the CMZ was suggested by lineage analysis in *Xenopus* [26] but was firmly demonstrated in medaka fish, using transplantation experiments and lineage analysis of single cells over a long period of time [26, 28]. Multipotent CMZ cells generate all retinal cell types in *Xenopus* [26]. In zebrafish, however, they do not give rise to rod photoreceptors [66, 94, 95]. Müller cells are the source of newly born rods throughout the lifetime of the zebrafish retina (described later in this review). Recent lineage analysis suggests that retinal stem cells in the CMZ preferentially undergo asymmetric cell divisions and hence maintain their overall population [96•]. Furthermore, evidence supporting this hypothesis was attained from the recent study using clonal and time-lapse analysis, which showed that after cell division one daughter cell remains as a retinal stem cell in the stem cell niche, while the other daughter cell is pushed centrally to become a retinal progenitor, eventually differentiating [97•].

The CMZ has now been molecularly well characterized in zebrafish and *Xenopus*. As mentioned in the “Introduction” section, CMZ cells are located in a spatial gradient resembling the temporal sequence of development [27, 66]. The different zones of the CMZ exhibit different combinations of transcription and post-transcription factors, signaling molecules, and cell cycle genes [27, 66, 98–106]. A comparative analysis of gene expression in the CMZ and during retinal development suggests that CMZ retinal stem cells originate from the neural retina–RPE border of the optic cup [101].

It has long been known that retinal injury in fish causes increased cell proliferation in the margin, suggesting that the CMZ contributes to retinal regeneration [107–111]. CMZ cells are however not the only cellular source of retinal repair in fish. Following ouabain-induced lesions, the distinct cone mosaic patterns in different regions of the regenerated retina suggested that newly born cones arise via two spatially and cellular distinct mechanisms [112]. The authors thus suggested that the CMZ is the major source of the regenerated peripheral retina but not of the central one. As described later in this review, Müller cells are the source of central retina regeneration in the fish. By single-cell lineage analysis in medaka, it was shown that CMZ stem cells maintain an asymmetric mode of cell division following retinal injury, as in the non-injured retina [96•]. Previous studies have suggested that rod photoreceptors are not generated from the CMZ of adult teleost fish [113, 114]. In agreement with this, it was recently shown in zebrafish following rod cell ablation, and using a lineage tracing analysis of the CMZ stem cell population, that these cells generate all retinal cell types except rod photoreceptors [115]. It remains unclear why there is an increased number of CMZ cells in a rod-degenerative model since they do not contribute to rod neurogenesis [115].

Although the RPE constitutes the major cellular source of regeneration following complete retinectomy in urodele amphibians, both the CMZ and the RPE (see below) participate in the replenishment of the tissue in post-metamorphic *X. laevis* [74, 75]. This different contribution of the CMZ appears to be highly variable even among *Xenopus* species. Indeed, in *Xenopus tropicalis*, the entire retina regenerates from the CMZ [78•]. In *Rana pipiens*, the CMZ is able to replenish specific cell types that were ablated by chemical treatment [116]. Whether this is the case in *X. laevis* has not been investigated. Interestingly, although there is no CMZ in mammals, the retinal margin was shown to have the potential to remain proliferative, attempting to generate a population of ganglion cells in a mouse model lacking RGC [117].

### From the RPE

The RPE is a monolayer of epithelial cells and has diverse functions such as forming a barrier between the choroid and the neural retina (blood–retina barrier), transporting nutrients, water, and ions from the choroid to the neural retina, phagocytosis of the photoreceptor outer segments, and recycling of the photosensitive opsin molecules for the visual cycle. As it is involved in the maintenance of normal structure and function of the retina, several retinal pathologies result from, or result in, the malfunction of the RPE [118].

RPE transdifferentiation into retina following retinectomy has been well described in the newt, an urodele amphibian [74, 118]. In the anuran *X. laevis*, the transdifferentiation potential of the RPE was thought to be lost after metamorphosis. It has however now been shown that it still occurs in the adult if the retinal vascular membrane (RVM), consisting of a basement membrane and numerous blood capillaries, is left in the ocular chamber during the retinectomy [75]. The process however differs greatly between the two amphibian species. The surgical removal of the newt retina results in proliferation of RPE cells, which regenerate both RPE and neural retina [119, 120]. In contrast, *X. laevis* RPE cells do not transdifferentiate at their original site. Instead, when the *X. laevis* retina is removed, while retaining the RVM, a subpopulation of RPE cells detach from the Bruch’s membrane, migrate to the RVM, proliferate, and form a neuroepithelium layer which generates all different retinal neurons and glial cells thereby regenerating the entire neural retina (Fig. 1d). RPE cells that remain at the original site renew the RPE itself [74, 75]. Cells that migrate were shown to express *Pax6* and acquire multipotency [121]. The presence of the RVM following retinectomy was critical, as the RPE failed to transdifferentiate if the RVM was removed. It was also suggested that the loss of contact with the Bruch’s membrane/choroid was critical, as it triggers the expression of *pax6* in RPE cells, allowing them to become multipotent and undergo migration and transdifferentiation in contact to the RVM [75, 121]. There are interesting questions that are now to be addressed, such as the following: What makes some of the RPE cells to stay whereas others migrate and transdifferentiate? What exactly are the presence of the RVM and the absence of the choroid doing to promote the regeneration process?

RPE transdifferentiation into all retinal cell types in adulthood appears to be specific to amphibians. It has indeed never been observed in fish. However, all amphibians are not relying on the RPE to regenerate their retina. As mentioned above, *X. tropicalis* regenerates its entire retina solely from the CMZ [78•]. Thus, in contrast to *X. laevis*, the RPE of *X. tropicalis* appears not to be involved in the regeneration process, even when the RVM remains in the ocular chamber following retinectomy. It is well recognized that in mammals too, RPE cells do not proliferate or self-renew upon injury. However, a study by Salero et al. demonstrated that RPE cells derived from human donors could be activated into multipotent cells that proliferated extensively and self-renewed in vitro. These multipotent cells could generate both neural and mesenchymal progeny in differentiation media that have been used previously to promote different cell types [122]. This study reveals a previously unappreciated plasticity of RPE cells that was thought to apply specifically to amphibians. Under appropriate conditions, mammalian RPE may thus also have the capacity to self-repair.

### From Müller Cells

Müller glial cells form the principle glial cells of the retina (constituting about 90% of the total glial cells and about 4–5% of the total retinal cells). They span the entire thickness of the retina from the GCL until the outer limiting membrane (OLM), the boundary between the cell bodies of the photoreceptors from their inner segments. Müller glial cell bodies are present in the INL, and their processes connect with all retinal neurons. Müller cells form a symbiotic metabolic relation with the adjacent photoreceptors, such that they provide the metabolites that are not synthesized within the photoreceptors and vice versa [123]. Müller cells are the last cells to be born during development and are in a quiescent post-mitotic state in *Xenopus*. Conversely, these cells in zebrafish continue to divide slowly and supply rod photoreceptors to the continuously growing retina [6, 57, 62, 124].

Regardless of the injury paradigm used to damage the retina (see above), Müller glia are the primary source of regenerated neurons in zebrafish [57, 58•, 66, 95, 124–129, 130•, 131–133]. Retinal functional recovery is however faster, with fewer histological errors, following selective damage that spares a population of neurons, as compared with extensive retinal damage [134]. Following injury, activated Müller cells undergo asymmetric division to self-renew and generate a retinal progenitor cell. The latter proliferates rapidly resulting in a cluster of progenitors which migrate to the area of damage and differentiate into the appropriate retinal cell type (Fig. 1e) [135, 136]. Indeed, although under normal physiological conditions Müller cells generate only rods, they are able to form all types of retinal neurons following retina damage [94]. It has recently been shown that Müller cells actually regenerate all retinal cell types regardless of which cells are initially damaged. Neurons in excess are then seeded into undamaged retinal layers [58•]. Upon cell cycle re-entry, Müller glial cells undergo interkinetic nuclear migration (INM), a process involving migration of nuclei along the apicobasal axis of the retina in phase with the cell cycle [91]. This migration was recently shown to be facilitated by the actin cytoskeleton and Rho-associated coiled-coil kinases (Rocks) and necessary for regeneration to occur [137•].

Reactive gliosis in mammals is beneficial to neurons through the release of a variety of neurotrophic factors that protect neurons from cell death. Prolonged gliosis, however, becomes detrimental and impedes Müller glial regenerative potential [138]. Zebrafish Müller glial cells also exhibit signs of reactive gliosis following retinal injury, prior to acting as stem cells [139]. Interfering with the Müller cell proliferative ability in zebrafish leads to persistent reactive gliosis, including hypertrophy and upregulation of Gfap, temporarily increasing Müller cell neuroprotective functions, but resulting in an inhibition of retinal regeneration [139]. This recent study highlights how modulating the balance between proliferative and non-proliferative gliosis can impact the balance between neuron protection and neuron replacement.

Apoptotic neurons in damaged retina produce factors, such as the secreted pro-inflammatory cytokine Tnfα, to initiate Müller glial proliferation [140]. Müller glial cells respond by re-expressing pluripotency genes such as *c-myc*, *nanog*, *nox2*, and *oct4* [141]. A recent transcriptomic analysis was made on fluorescence-activated cell sorting (FACS)-sorted Müller glia following retinal lesion [142]. This study revealed several other categories of genes/signaling rapidly upregulated in reactive Müller cells, such as nuclear factor-κB (NF-κB) signaling, prostaglandin metabolism, and clock genes. These represent potential novel candidates involved in Müller cell-dependent regeneration of retinal neurons. Activated Müller glial cells themselves are a source of growth factors and cytokines that drive and amplify their own proliferative activity [143•]. Several regeneration-associated signaling pathways activated in reactive Müller cells and required for their proliferation were identified in the last few years, such as Wnt/β-catenin, MAPK–Erk, PI3K/Akt, and Jak–Stat signaling (reviewed in detail in [95, 144]). On the other hand, inhibitory effects of some pathways such as TGFβ were reported [95]. In support with this, it was recently shown that inhibition of the TGFβ signaling pathway results in increased Müller cell proliferation following retinal degeneration [145].

Ascl1 is a critical proneural basic helix–loop–helix (bHLH) transcription factor upregulated in Müller cells following retinal damage and required for their proliferation during retinal regeneration [125, 146]. The different regulatory mode of this gene between mammals and fishes was proposed as one of the reasons underlying the different regenerative ability between these species [147]. It was indeed shown in young mice that forced expression of *Ascl1* in Müller glia could stimulate their capacity for retinal regeneration. Another bHLH factor, Atoh7, was recently shown to be sufficient to trigger a proliferative and neurogenic response of quiescent Müller cells in medaka fish [148].

A key question is whether Müller cell-derived neurons can restore their original connectivity patterns. It was recently shown in zebrafish that regenerated bipolar cells following retinal damage can achieve functional integration in the retina [149•, 150]. However, stereotypic dendritic wiring patterns are not fully re-established. The authors thus propose that regenerated bipolar cells may exhibit visual response properties that do not resemble those of the original population. Regarding horizontal cells, it was shown that following ablation of UV cones, H3 horizontal cells that normally prefer UV cones re-establish contact with newly regenerated UV cones [151]. This study thus demonstrates that the preference of synaptic partners is maintained. This however is true only if lost cones are rapidly replaced, since misconnections are observed if regeneration is delayed.

Many regeneration studies performed in *Xenopus* involved the removal of the complete retina, thereby preventing the analysis of resident Müller cells during the regenerative process. Although retinal precursor cells were found at the wound site after partial retinal resection in pre-metamorphic *X. laevis*, their Müller cell identity was not investigated [80]. In a model of conditional rod cell ablation, Müller cell hypertrophy was observed suggesting the occurrence of reactive gliosis. Yet, in contrast to the fish situation, no proliferative Müller cells were observed [152]. Recently, however, we were able to show in a similar model of conditional rod cell ablation that *X. laevis* Müller cells are able to re-enter into the cell cycle and contribute to the regeneration of retinal neurons (our unpublished data). Interestingly and unexpectedly, we found that the extent of cell cycle re-entry appears dependent on the age of the animal, being very limited in young tadpoles compared to pre-metamorphic, post-metamorphic, or adult specimen. This may be one element underlying the apparent different results reported so far on Müller cell response in a damaged *X. laevis* retina. *Xenopus* is thus able to mobilize CMZ cells, RPE cells, or Müller cells according to the injury paradigm.

## Conclusions

Together, these few illustrations highlight that zebrafish and *Xenopus* models are emerging as key organisms for retinal disease modeling to provide a deeper understanding of the pathogenesis of the disease, identify potential causative genes, and screen for novel drugs. It is expected that CRISPR-Cas9 technology, as a genome-editing tool, will further facilitate the development of such models that mimic retinal degenerative diseases.

The regenerative abilities of the zebrafish and *Xenopus* are truly remarkable. Species-specific differences in the mode of regeneration are intriguing and deserve further investigations, to better understand the evolutionary constrains behind neural tissue regeneration. Studies involving comparative evolutionary approaches could bring insights into the molecular cues that either sustain or prevent neural cell replacement and therefore help to design and develop therapies to stimulate retinal regeneration in mammals.
